# Survivin in Insulin-Like Growth Factor-Induced Resistance to Lapatinib in Head and Neck Squamous Carcinoma Cells

**DOI:** 10.3389/fonc.2019.00013

**Published:** 2019-01-23

**Authors:** Christine E. Lehman, Rolando E. Mendez, Michael I. Dougherty, Amir Allak, Oluwayemisi L. Adejumo, Linnea E. Taniguchi, Ashraf Khalil, Daniel G. Gioeli, Mark J. Jameson

**Affiliations:** ^1^Division of Head and Neck Oncologic and Microvascular Surgery, Department of Otolaryngology–Head and Neck Surgery, University of Virginia Health System, Charlottesville, VA, United States; ^2^Department of Biochemistry, National Liver Institute, Menoufia University, Shibin al Kawm, Egypt; ^3^Department of Microbiology, Immunology and Cancer Biology, University of Virginia Health System, Charlottesville, VA, United States

**Keywords:** survivin, IGF1R, lapatinib, EGFR, HNSCC, resistance

## Abstract

Epidermal growth factor receptor (EGFR) inhibitors have limited efficacy in head and neck squamous cell carcinoma (HNSCC) due to various resistance mechanisms, such as activation of the insulin-like growth factor-1 receptor (IGF1R), which initiates pro-survival signaling. Survivin, a member of the inhibitor of apoptosis proteins family, is expressed at relatively high levels in malignant tissues and plays a role in cell division. Expression of survivin in tumors has been shown to correlate with poor prognosis due to chemotherapy resistance and anti-apoptotic behavior. We previously demonstrated that activation of the IGF1R reduces sensitivity to EGFR-tyrosine kinase inhibitors (TKIs) via reduced apoptosis suggesting a role of survivin in this process. This study evaluates the role of survivin in IGF1R-mediated lapatinib resistance. Using HNSCC cell lines FaDu and SCC25, survivin expression increased and lapatinib sensitivity decreased with IGF1R activation. Further, these effects were reversed by the survivin inhibitor YM-155. Conversely, survivin expression and lapatinib sensitivity were unchanged with IGF1R activation in UNC10 cells. YM-155 enhanced the inhibitory effect of lapatinib on UNC10 cells, regardless of activation of the IGF1R. These results demonstrate that enhanced survivin expression correlates with IGF1R-mediated lapatinib resistance in HNSCC cells and suggest that regulation of survivin expression may be a key mechanistic element in IGF1R-based therapeutic resistance. Combinatorial treatment with survivin antagonists and EGFR-TKIs warrants further investigation.

## Introduction

Head and neck squamous cell carcinoma (HNSCC) constitutes ~3% of all malignancies with over 50,000 new cases diagnosed in the United States alone (1). In stage III and IV HNSCC, 5-year survival rates are below 50% and have not substantially improved in the past several decades (2). Epidermal growth factor receptor (EGFR) tyrosine kinase inhibitors (TKIs) have potential as a molecular targeted therapy for HNSCC because more than 90% overexpress the EGFR (3, 4). However, phase II clinical trials with single-agent targeted EGFR inhibitors yielded response rates below 15% despite EGFR expression and inactivation (5, 6). These findings suggest a compensatory mechanism that promotes cell survival despite EGFR inhibition, resulting in therapeutic resistance. A better understanding of this resistance mechanism has the potential to lead to more effective targeted therapy for HNSCC.

Survivin is a 16.5 kDa protein that is a member of the inhibitor of apoptosis proteins (IAP) family. In normal adult tissue, survivin is only expressed at trace levels, primarily in the thymus and placenta. However, survivin is expressed at relatively high levels in malignant tissues (7). Survivin plays a role in cell division and is increased during the G2/M phase of the cell cycle and plays a protective role to the microtubules of the mitotic spindle (8). Overexpression of survivin causes persistent replication in the face of an errant chromosome and such a phenomenon might contribute to the progression of cancer (9). Survivin inhibits apoptosis; this was thought to occur via binding to active caspases (10) but recent data shows no consensus on its mechanism of action (11). Expression of survivin in tumors has been shown to correlate with poor prognosis (12, 13) related to resistance to chemotherapy, higher rate of recurrence, and anti-apoptotic behavior (14).

We have previously demonstrated that activation of the insulin-like growth factor-1 receptor (IGF1R) reduces sensitivity to EGFR-TKIs in HNSCC cell lines via reduced apoptosis (3, 15). This effect is associated with increased Akt activity, but the downstream mediators have not been defined. Overexpression of survivin has been associated with reduced apoptosis in HNSCC (16). In breast cancer, the EGFR/HER2 TKI lapatinib has been shown to reduce levels of intracellular survivin via a ubiquitin-mediated proteasome degradation mechanism (17). Survivin expression has also been shown to increase with exposure to insulin-like growth factor-1 (IGF-1) in prostate cancer (18). Therefore, it is possible that IGF-induced survivin expression contributes to the EGFR-TKI resistance we have previously demonstrated.

In the present report, we explore the role of survivin in IGF1R-mediated lapatinib resistance in HNSCC cell lines *in vitro*. If IGF-stimulated survivin expression confers resistance to lapatinib, co-treatment with a suppressor of survivin expression, such as YM-155 which inhibits survivin gene promoter activity, may have potential to improve therapeutic outcomes for patients with HNSCC.

## Materials and Methods

### Reagents

des[1-3]IGF-1 (desIGF1), an N-terminally truncated form of insulin-like growth factor-1, was obtained from Cell Sciences (Canton, MA). AlamarBlue, Dulbecco Modified Eagle Medium (DMEM)/F-12 culture medium and fetal bovine serum (FBS) were obtained from Invitrogen (Carlsbad, CA). YM-155 was obtained from Chemietek (Indianapolis, IN) and lapatinib from LC Laboratories (Woburn, MA). Anti-β-actin and anti-survivin antibodies were obtained from Cell Signaling Technology (Beverly, MA). Lapatinib was dissolved in dimethyl sulfoxide (DMSO) and diluted as appropriate in water. DMSO concentration did not exceed 0.1% in tissue culture experiments; at this maximal concentration, DMSO alone had no impact on cell number as assessed by alamarBlue. All experiments involving lapatinib were controlled using treatment with vehicle which contained an equivalent concentration of DMSO.

### Cell Culture

SCC25 and FaDu cells were obtained from ATCC (Manassas, VA). UNC10 cells were kindly provided by Dr. Wendell Yarbrough (Yale University, New Haven, CT). Cell line identities were confirmed by DNA fingerprinting (University of Arizona). Cells were cultured in DMEM/F-12 medium with HEPES supplemented with 5% FBS and 400 ng/mL hydrocortisone, and maintained in a 37 C humidified incubator with 5% CO_2_. All cell lines were routinely tested and found to be free of mycoplasma contamination using MycoAlert (Lonza, Allendale, NJ).

### Immunoblot

Cells were cultured in either 6 or 10 cm plates. Cells were treated with 5 μM lapatinib for 2 h followed by 10 nM desIGF1 for 24 h prior to lysis. In some experiments, cells were pre-treated with 100 nM YM-155 for 24 h. Cells were washed with ice-cold phosphate-buffered saline (PBS) with 2 mM sodium orthovanadate, then scraped and collected in PBS containing 2 mM sodium orthovanadate, and pelleted at 4,000 rpm for 5 min. The pellet was treated with lysis buffer (50 mM HEPES, 10 mM Na pyrophosphate, 100 mM NaF, 4 mM EDTA, 1% Triton X-100, 0.1 M PMSF 0.2 M sodium orthovanadate, 10 mM benzamide and 1 mg/ml of aprotinin, leupeptin and pepstatin), vortexed, and incubated on a rotator at 4°C for 30 min. Protein-containing solution was separated from cellular debris by centrifugation. Protein concentration was determined using Pierce BCA Protein Assay Kit. 12% SDS-polyacrylamide gels were loaded with at least 20 μg of lysate followed by electrophoresis at 100 V for 20 min and 175 V for 50 min. The protein was transferred to PVDF membrane (Immobilon-FL Transfer Membrane, Millipore, Billerica, MA) at 15 V for 45 min in a semi-dry transfer apparatus. The membrane was blocked with 0.1% casein in 1x PBS and incubated with primary antibody overnight at 4°C. The membrane was washed in 1x Tris buffered saline with 0.05% Tween 20 (TBST), incubated with secondary antibody (0.1% casein in 1x PBS containing 0.05% Tween 20 and 0.01% SDS) for 45 min, washed in TBST, and rinsed with PBS. Immunoblots were analyzed using the Odyssey imaging system (LICOR Biosciences, Lincoln, NE).

### Cell Viability Assay

Five thousand cells in 100 μL of DMEM/F12 containing 0.5% FBS were added to a 96-well plate. Cells were grown for 24 h, then fresh medium containing 0.5% FBS was added with appropriate inhibitor(s) for 2 h, followed by stimulation with desIGF1 for 72 h; each treatment was performed in at least triplicate. Ten microliter of alamarBlue (Invitrogen) was added to each well according to the manufacturer's protocol. Cells were incubated for 3–4 h at 37 C and the fluorescence at 540 nm was recorded using a Synergy 2 multi-mode microplate reader (BioTek, Winooski, VT). Net fluorescence for each condition was normalized to uninhibited/unstimulated controls and averaged across replicates. GI_50_ values for lapatinib were calculated by using a non-linear regression model with GraphPad Prism 7.0 software (GraphPad Software, Inc., San Diego, CA).

## Results

### Effect of Lapatinib on HNSCC Cell Viability

**Figure 1:**
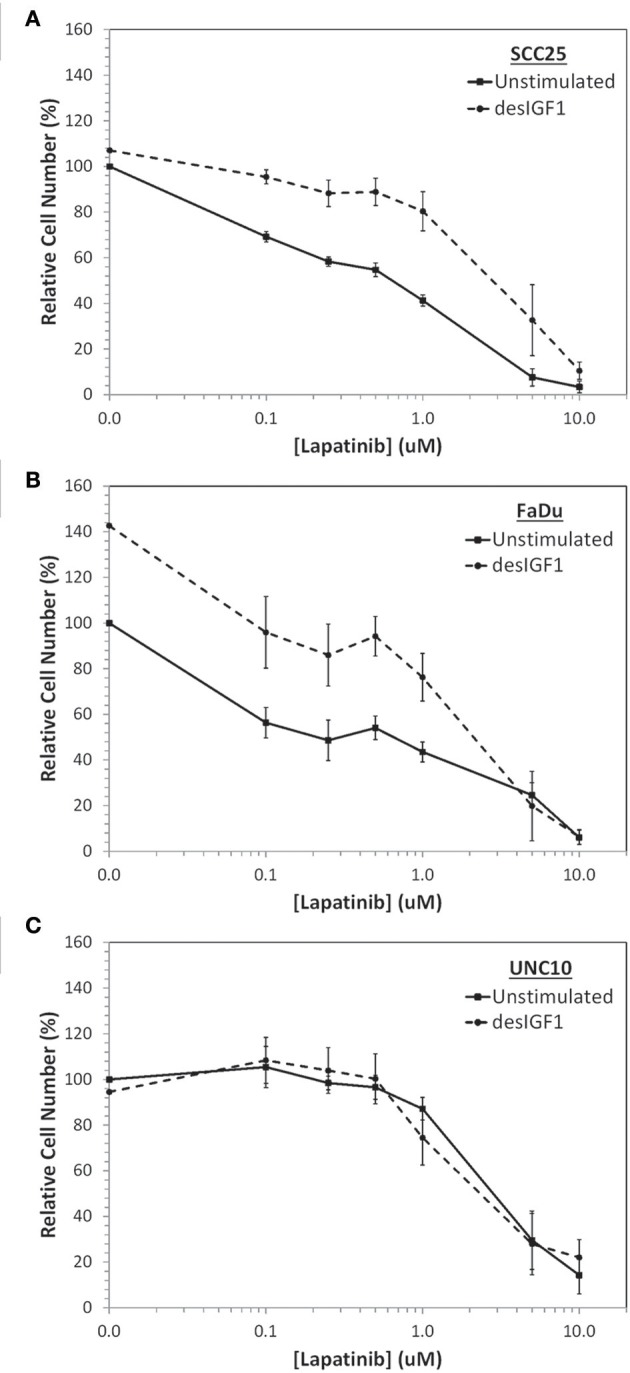
Inhibition of cell viability by lapatinib in HNSCC cell lines and rescue with IGF stimulation. Dose-dependent effect of lapatinib ± 10 nM des[1-3]IGF-1 (desIGF1) on **(A)** SCC25, **(B)** FaDu, and **(C)** UNC10 cells as assessed by alamarBlue. Graphs show relative cell number (as determined by net fluoresence) ± SEM as a percentage of uninhibited/unstimulated cells for at least 3 independent experiments.

SCC25, FaDu, and UNC10 cells were plated onto 96-well plates and treated with various concentrations of lapatinib or vehicle for 2 h followed by 10 nM desIGF1 or vehicle for 72 h. Using alamarBlue to examine viability, all 3 cells lines demonstrated dose-dependent growth inhibition in response to lapatinib treatment (Figure 1). For SCC25 and FaDu cells, the dose-response curve shifts to the right with addition of desIGF1 indicating a protective effect. The GI_50_ for lapatinib was ~0.50 and 0.35 μM for SCC25 and FaDu cells, respectively. These shifted to 3.15 and 2.10 μM, respectively with IGF treatment (Figures 1A,B), representing a 5–7-fold reduction in lapatinib sensitivity. As shown in Figure 1C, lapatinib has a GI_50_ of 2.90 μM in UNC10 cells, indicating much less sensitivity than the other two HNSCC cell lines. Addition of desIGF1 to UNC10 cells had no effect on lapatinib sensitivity. Based on these dose-response studies, SCC25 and FaDu cells are designated as “IGF-responsive” with respect to lapatinib sensitivity, while UNC10 cells are designated as “IGF-non-responsive.”

### Molecular Characterization of Lapatinib and EGF in HNSCC Cells

**Figure 2:**
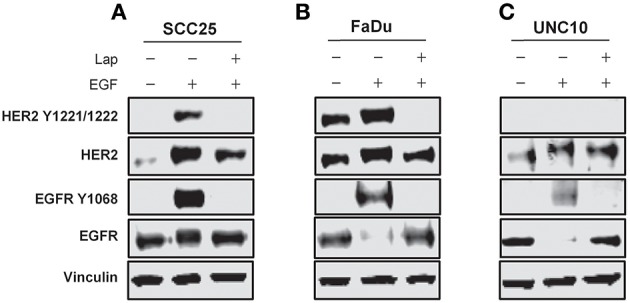
Molecular characterization of HNSCC cell lines stimulated with EGF or inhibited with Lapatinib. SCC25 **(A)**, FaDu **(B)**, and UNC10 **(C)** cells were treated with 5 μM lapatinib (Lap) and/or EGF as shown. Whole cell lysates were subjected to immunoblot for HER2, EGFR, p-HER2, p-EGFR, and Vinculin. Representative immunoblots are shown representing at least 2 independent experiments.

SCC25, FaDu, and UNC10 cells were stimulated with EGF for 10 min in the presence or absence of lapatinib and assessed by immunoblot for expression of EGFR, HER2 and respective phosphorylated EGFR and HER2 (Figure 2) to examine basal levels and determine the ability of EGFR and HER2 to be stimulated in each cell line. Each cell line demonstrated basal EGFR and HER2 expression, to varying degrees. Further, each cell line demonstrated stimulation of EGFR Y1068 when treated with EGF and inhibition of this stimulation by lapatinib treatment. HER2 Y1221/1222 was also stimulated by EGF in SCC25 and FaDu, however, no phosphorylation was observed in UNC10. While HER2 Y1221/1222 was not stimulated by EGF in UNC10 cells, Lapatinib effectively inhibited EGFR Y1068 in all cell lines and effectively inhibited HER2 Y1221/1222 in SCC25 and Fadu. Therefore, since Lapatinib effectively inhibits HER2 and desIGF1 still rescues cell growth during this inhibition, HER2 is not involved in a pathway of survival under desIGF1 treatment.

### Effect of Desigf1 and Lapatinib on Survivin Expression

**Figure 3:**
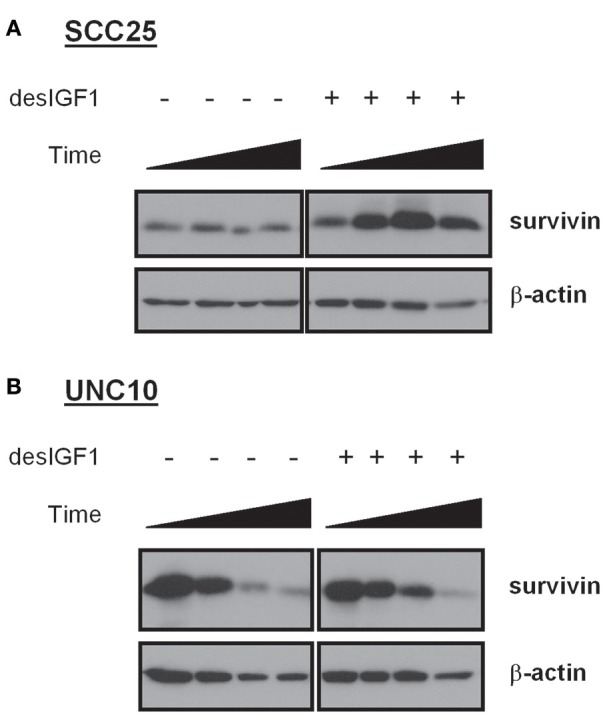
Survivin expression in HNSCC cell lines and effect of IGF stimulation. SCC25 **(A)** and UNC10 **(B)** cells were treated with vehicle or 10 nM des[1-3]IGF-1 (desIGF1) for 5 min, 4, 24, and 48 h. Whole cell lysates were collected and subjected to immunoblot with the indicated antibodies.

After 24 h of serum starvation, SCC25 and UNC10 cells were cultured in serum free medium for 48 h with or without desIGF1. Cells were harvested at 5 m, 4, 24, and 48 h and assessed for survivin expression by immunoblot as shown in Figure 3. Without stimulation, SCC25 cells maintained stable survivin levels over the 48 h period. When SCC25 cells were stimulated with desIGF1, survivin levels increased, peaking at 24 h (Figure 3A). In contrast, stimulation of UNC10 cells with desIGF1 did not increase survivin expression (Figure 3B). These findings demonstrate that SCC25 cells, which exhibit IGF-induced lapatinib resistance, have lower survivin expression that is inducible by IGF1R activation. Further, UNC10 cells are IGF-non-responsive in that survivin is not augmented by IGF stimulation.

**Figure 4:**
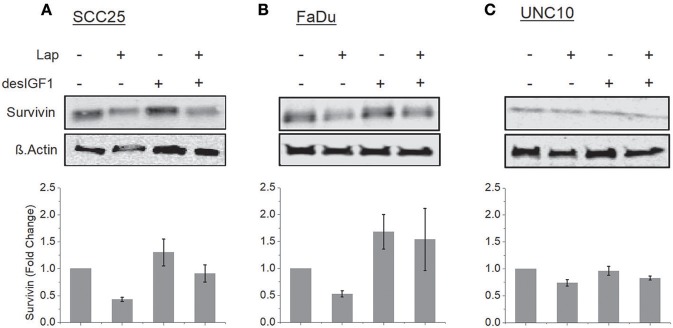
Lapatinib inhibition of survivin expression is overcome by IGF stimulation. SCC25 **(A)**, FaDu **(B)**, and UNC10 **(C)** cells were treated with 5 μM lapatinib (Lap) and/or 10 nM des[1-3]IGF-1 (desIGF1) as shown. Whole cell lysates were subjected to immunoblot for survivin. Representative immunoblots are shown above and pooled quantifications of at least 3 independent experiments are shown below as fold change relative to vehicle-treated controls. Data are represented as the average relative band density ± SEM.

SCC25, FaDu, and UNC10 cells were treated for 24 h with lapatinib in the absence and presence of desIGF1 and assessed by immunoblot for survivin expression. In SCC25 cells, lapatinib caused 57% reduction in survivin expression while desIGF1 increased survivin expression by 30% compared to untreated controls. When desIGF1 was combined with lapatinib, survivin expression was maintained near basal level (Figure 4A). A similar pattern was seen in FaDu cells (Figure 4B). Lapatinib decreased survivin level by 48% while desIGF1 increased expression 65%; combination treatment resulted in sustained elevation of survivin expression despite the presence of lapatinib. Thus, in HNSCC cell lines that demonstrate IGF-responsiveness, IGF1R activation reverses the survivin reduction caused by lapatinib treatment. In UNC10 cells, addition of lapatinib decreased survivin levels by 26% while treatment with desIGF1 had no effect on basal survivin expression (96% of control). Combination treatment yielded survivin levels similar to untreated cells (85% of control) (Figure 4C).

### YM-155 Inhibits Survivin Expression and Reverses IGF-1 Induction

**Figure 5:**
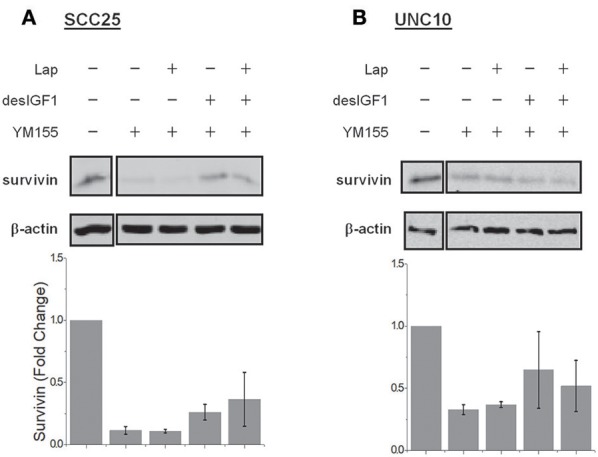
YM155 inhibits survivin expression and reverses IGF-1 induction. SCC25 **(A)** and UNC10 **(B)** cells were treated with 100 nM YM155 for 24 h to inhibit survivin expression. Cells were then treated with 5 μM lapatinib (Lap) and/or 10 nM des[1-3]IGF-1 (desIGF1) for 24 h as shown. Representative immunoblots are shown above and pooled quantifications of 2 independent experiments are shown below as fold change relative to vehicle-treated controls. Data are represented as the average relative band density ± SEM.

YM-155 is a small-molecule survivin inhibitor. Its effects are mediated by suppression of the survivin gene promoter (19). SCC25 cells treated with YM-155 demonstrated decreased survivin levels (Figure 5A). YM-155 blocked increased survivin expression upon treatment with desIGF1 in both the absence and presence of lapatinib. YM-155 had similar effects in UNC10 cells (Figure 5B). These data demonstrate that YM-155 effectively reduces basal and IGF-induced survivin expression.

**Figure 6:**
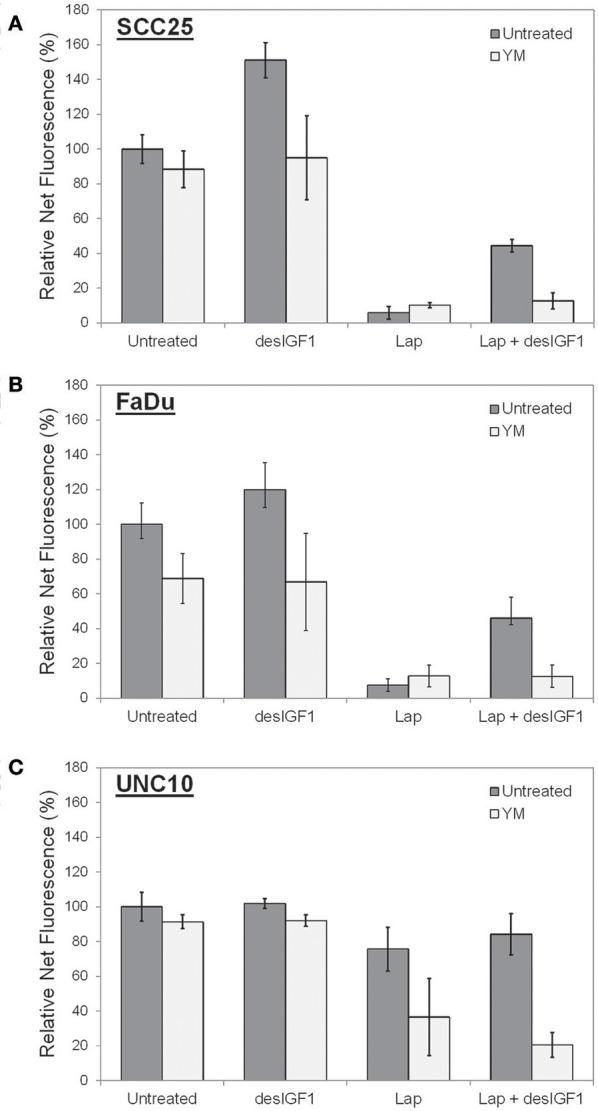
YM155 reverses IGF rescue of HNSCC cells from lapatinib growth inhibition. Proliferation of **(A)** SCC25, **(B)** FaDu, and **(C)** UNC10 cells was assessed by alamarBlue assay. Cells were treated with 5 nM YM155 (YM), 5 μM lapatinib (Lap), and/or 1 nM des[1-3]IGF-1 (desIGF1) for 72 h. Graphs show average net fluorescence (a surrogate for cell number) as a percentage of uninhibited/unstimulated cells for 3 independent experiments ± SEM.

Cell viability assays were performed using alamarBlue to assess the effect of YM-155 on IGF-induced lapatinib resistance in SCC25 and FaDu cells. As UNC10 do not demonstrate IGF-induced lapatinib resistance, they were included as a negative control. A low dose of YM-155 (5 nM) was used in these studies in an attempt to minimize the independent impact on proliferation and to minimize off-target effects. SCC25 cells treated with 5 nM YM-155 showed no significant change in fluorescence, a surrogate marker of viability (Figure 6A). DesIGF1 treatment increased viability by 51%; this effect was blocked by addition of YM-155. Cell viability was reduced by 95% with lapatinib treatment and was further unchanged by addition of YM-155. With desIGF1 stimulation, SCC25 cell viability was reduced by only 56% with lapatinib; this rescue was reversed by YM-155. Similar results were obtained with FaDu cells (Figure 6B), except that YM-155 alone caused a 32% reduction in cell viability. As in SCC25 cells, YM-155 treatment of FaDu cells inhibited desIGF1-stimulation of cell viability and desIGF1-induced lapatinib resistance. UNC10 cells demonstrated no response to YM-155 treatment or desIGF1 stimulation (Figure 6C). Lapatinib caused a modest reduction in cell viability (25%) with no significant rescue by desIGF1. Interestingly, the addition of YM-155 to lapatinib treatment induced a 64% decrease in cell viability despite the resistance of this cell line to lapatinib treatment. Addition of desIGF1 did not reverse this effect of combined lapatinib and YM-155. It is interesting that, despite the apparent low level of survivin expression in UNC10s, addition of YM-155 further reduces survivin in these cells (Figure 5), and that decrease is associated with improved effectiveness of lapatinib (Figure 6C, lapatinib with and without YM-155). This effect is not overcome by activation of the IGF1R (Figure 6C, lapatinib with desIGF1 with or without YM-155). These data strongly suggest that, in UNC10 cells, despite its apparent low level, survivin may be a key predictor of responsiveness to lapatinib. They also reinforce the notion that the ability of IGF1R signaling to increase survivin levels is key to the resistance phenomenon.

## Discussion

Given their very different GI_50_ values in cell viability assays, SCC25 and FaDu cells are considered sensitive to lapatinib while UNC10 cells are considered resistant. In sensitive SCC25 and FaDu cells, basal survivin expression is lower and is more dramatically reduced in the presence of lapatinib. In resistant UNC10 cells, basal survivin expression is high and shows limited reduction in response to lapatinib treatment. This suggests the possibility that survivin expression may be a predictive marker of lapatinib sensitivity, and perhaps sensitivity to other EGFR-TKIs. However, a broader assessment of tumors would be necessary to make a definitive correlation.

Activation of the IGF1R reduces the sensitivity of SCC25 and FaDu to lapatinib treatment. This is similar to the effect on gefitinib sensitivity that we have demonstrated (3), and we presume occurs via pro-survival signaling that reduces EGFR-TKI-induced apoptosis as previously reported (3). In the present study, we demonstrate that this rescue effect is associated with an induction of survivin expression in response to IGF1R activation. Conversely, IGF1R activation did not substantially increase survivin expression in UNC10 cells and also had no impact on lapatinib sensitivity. This demonstrates that IGF1R-mediated resistance correlates with the ability of the IGF stimulation to increase survivin levels, implying that survivin may be a necessary element to the rescue phenomenon.

These observations point to a role for survivin in determining the cellular response to lapatinib and possibly other EGFR-TKIs. Several studies have connected sensitivity to EGFR-TKIs and survivin in non-small cell lung cancer. In one model system, survivin overexpression was shown to attenuate response to gefitinib *in vitro* and *in vivo* (20). A subsequent study involved development of a gefitinib-resistant cell line which was noted to have elevated survivin levels compared to its gefitinib-sensitive clone, suggesting a role of survivin in acquired resistance to EGFR-TKIs (21). A clinical study found that survivin mRNA levels in blood were strongly associated with a poor response to EGFR-TKI treatment in patients with non-small cell lung cancers (22), indicating that survivin expression may be a predictor of intrinsic resistance to EGFR-TKIs.

YM-155 blocked IGF-stimulated survivin expression in both the absence and presence of lapatinib. In SCC25 and FaDu cells, YM-155 treatment inhibited IGF-stimulation and IGF1-mediated lapatinib resistance. This implies an important role for survivin in IGF1R-induced proliferation of both uninhibited and lapatinib-treated cells. In prior studies, increases in cell number in IGF-stimulated cells were due to the anti-apoptotic effect of IGF1R (3). Other studies have confirmed a link between survivin and anti-apoptotic effects of IGF1. In prostate cancer, IGF-1 induced survivin expression via activation of the mTOR/p70S6K axis, leading to increased translation of pre-existing survivin mRNA; this process was inhibited by introduction of a p70S6K siRNA and by the mTOR inhibitor rapamycin (18). IGF-1 also induced survivin in renal cancer cells, and this effect was associated with proliferation (23). In non-small cell lung cancer, IGF-1 treatment of erlotinib-inhibited cells increased IGF1R/EGFR heterodimerization, leading to mTOR-mediated synthesis of EGFR and survivin, which counteracted the antiproliferative effects of Erlotinib (24).

Treatment of UNC10 cells with lapatinib or YM-155 alone had a very limited effect on cell viability. However, treatment with both drugs simultaneously resulted in increased growth inhibition, implying that the inherent resistance mechanism present in UNC10 cells was overcome. The growth inhibitory effect of combined lapatinib and YM-155 was not reversed by IGF1R activation, indicating that this inducible resistance mechanism was blocked by the combination in UNC10 cells as it was in the other two cell lines. Thus, some inherent EGFR-TKI resistance in HNSCC may be related to survivin expression and may be reversible with anti-survivin therapy.

The present data suggest that survivin is an important mediator of acquired and intrinsic resistance to lapatinib in HNSCC. Survivin is already a promising biomarker for poor clinical outcomes in other malignancies such as in prostate and endometrial cancer (25, 26). The latter study demonstrated YM-155-induced apoptosis in 16 endometrial cancer cell lines. The clinical relevance of the IGF1R/Survivin signaling axis is a very pertinent question. Querying The Human Protein Atlas demonstrates that 5-year survival of HNSCC decreases from 50 to 35% if Survivin levels are high (log-rank *p* = 0.038) (27). Further, while the number of cases with alteration in the TCGA provisional dataset was too low to demonstrate significance, median disease-free survival of HNSCC dropped from 61.07 to 27.89 months with an alteration in the Survivin gene (28–30). These data suggest that survivin may play an important negative prognostic role in HNSCC and could well-predict responsiveness to/success of therapy. Given that we have previously identified high levels of basal IGF1R activity in human HNSCC tumors (3) and that we presently correlate IGF1R signaling and survivin expression in some cell lines, there could be a direct connection between IGF1R signaling and poorer survival through survivin expression. In HNSCC, further studies are warranted to examine the efficacy of co-administration of EGFR-TKIs and survivin inhibitors *in vivo*, noting the impact on models of both inherent and acquired resistance to EGFR-TKIs.

Survivin expression in HNSCC may be an important determinant of sensitivity to lapatinib and other EGFR-TKIs. Regulation of survivin expression by the IGF1R plays a role in IGF1R-mediated resistance to EGFR-TKIs. Inhibition of survivin expression reverses both inherent and IGF1R-mediated lapatinib resistance in selected cell lines. Thus, survivin inhibition may have utility in the therapeutic approach to HNSCC that exhibits inherent or acquired resistance to EGFR-TKIs.

## Author Contributions

CL and RM were involved in experimental work, experimental planning and analysis, and manuscript preparation. MD, AA, OA, LT, and AK were involved in experimental work. DG and MJ supervised the findings of the work, and were involved in experimental planning, analysis, and manuscript preparation.

### Conflict of Interest Statement

The authors declare that the research was conducted in the absence of any commercial or financial relationships that could be construed as a potential conflict of interest.
